# Academic ophthalmology in the post-COVID-19 era

**DOI:** 10.1080/10872981.2020.1830680

**Published:** 2020-10-05

**Authors:** Victor Eduardo Reviglio, Matias Osaba, Pablo Chiaradia, J. Fernando Arevalo

The current pandemic triggered by coronavirus disease 2019 (COVID-19) has created an unprecedented global social, economic, healthcare, and educational crisis. Albert Einstein stated that ‘crisis is the greatest blessing for people and nations, because the crisis brings progress.’

Historically, when humanity is confronted with a crisis, significant scientific, societal and technologic advances have followed. Examples from the past include the invention of penicillin and more recently, robot-assisted surgery, and ophthalmic devices in ophthalmology that allow automated measurements thereby reducing person-to-person interaction potentially decreasing the risk of transmission.

Increased globalization coupled with technological advances have allowed ophthalmologists and vision scientists to incorporate knowledge, integrate techniques and simultaneously expand our professional field worldwide. Videoconferencing is an extremely efficient and effective tool at academic medical institutions for enhancing the teaching and learning process [1]. Videoconferencing allows real-time communication, increasing productivity and allowing timely dissemination of clinical and scientific information irrespective of the geographic distances and time differences. Hence, scientific conversations can turn into impromptu roundtable discussions and there is exchange of scientific concepts in an unstructured but productive manner. This information revolution is due to advances in computing ability that increased data transmission and, provided a powerful and interactive means for sharing clinical and scientific information. However, the majority of academia has been slow to adopt videoconferencing and other modes of digital communication. Person-to-person skill training programs in medicine, public conferences, and printed books remain the gold standard in medical and scientific education. However, newer technologies have provided an effective adjunct to continue teaching and training students globally during this period of nationwide lockdowns. As the COVID-19 pandemic took hold, digital communications increased; scientists, biochemists, medical doctors, and academic institutions are now interconnected in a new global real-time information exchange network. For example, published scientific studies are analyzed, applied, or criticized globally within a few hours, carrying both risks and benefits [2].Effectively, the current pandemic crisis has highlighted opportunities for academia to redesign aspects of clinical education and scientific training that may change the way we teach post-COVID-19.

Although telemedicine has been implemented in dangerous and critical situations since the Gulf War (1991), inter-hospital medical and teleconferencing sessions have only increased in recent years [3,4].Our experience as ophthalmologists may well be an impetus for some permament changes to our profession [5]. This crisis allowed us to generate new measures for telemedicine video conferencing patient care, diagnosis and treatment. However, after the World Health Organization declared a global emergency, academic and scientific information was exchanged by participation in hundreds of webinars involving thousands of ophthalmologists worldwide, allowing unprecedented advances in ophthalmology over a short period. Professors around the world found themselves conducting virtual webinars, research lab meetings or academic seminars, communicating with their colleagues, residents or students over social media platforms, providing distance education to students affected by the closure of ophthalmological departments and academic units. Videoconferencing of a professor or surgeon instructor observing a resident or students in an online setting are common place and peers observe both audiences to provide a positive learning feedback loop to both parties.

As medical schools have suspended their clinical rotations, the normal residency training programs have severely disrupted student education. Thus, telemedicine has become vital for residency evaluation and follow up [6,7].Online healthcare resources and academic webinars have been helpful for complementing medical education in clinical and surgical procedures for residents.

However, there are two important questions regarding virtual training and free access to information. First, could virtual education replace some traditional modes of education, including training in clinical assessment and surgical procedures or the development of specific skills required by subspecialists? Second, is the temporary free access to academic libraries and scientific journals a unique and transitory experience, or will it be a social and scientific objective allowing, perhaps greater cognitive growth by maintaining free access?

These changes that have been precipitated by the pandemic emphasize the importance of interactive web platforms, which provide a means of virtual clinical and surgical learning, practice for multiple choice tests, and discussions of complex cases during grand rounds.

Therefore, in these times of lockdown due to the SARS-CoV-2, the advancement of scientific and academic progress moves our medical community towards a new ophthalmic era that we are all collectively changing in real time.

‘We cannot expect things to change if we keep doing the same things’

The crisis according to Albert Einstein (1955)

## References

[1] Flodgren G, Rachas A, Farmer AJ, et al. Interactive telemedicine: effects on professional practice and health care outcomes. Cochrane Database Syst Rev. 2015;2015:CD002098. 10.1002/14651858.CD002098.pub2 PMC6473731 26343551

[2] Adhikari SP, Meng S, Wu YJ, et al. Epidemiology, causes, clinical manifestation and diagnosis, prevention and control of coronavirus disease (COVID-19) during the early outbreak period: a scoping review. Infect Dis Poverty. 2020;9(1):29. 32183901 10.1186/s40249-020-00646-x PMC7079521

[3] Kane CK, Gillis K. The use of telemedicine by physicians: still the exception rather than the rule. Health Aff (Millwood). 2018;37(12):1923‐1930. 10.1377/hlthaff.2018.05077 30633670

[4] Kelton DK, Szulewski A, Howes D. Real-time video telemedicine applications in the emergency department: a scoping review of literature. CJEM. 2018;20(6):920‐928. 10.1017/cem.2017.382 28829008

[5] American Academy of Ophthalmology. Your COVID-19 experience drives academy efforts on your behalf. [cited 2020 422].Available from: https://www.aao.org/about/governance/academy-blog/post/your-covid-19-experience-drives-academy-efforts

[6] Jin M, Jackson C, AlAbdulKader AM, et al. ACMQ student, resident, and fellow section (SRF) COVID-19 resident and fellow survey. Am J Med Qual. 2020;35(3):289–2. 32418439 10.1177/1062860620926271 PMC7281911

[7] Bauchner H, Sharfstein J, Bold A. Response to the COVID-19 pandemic: medical students, national service, and public health [published online ahead of print, 2020 Apr 8]. JAMA. 2020. DOI:10.1001/jama.2020.6166 32267488

